# Father-Teen Talks about Sex and Teens’ Sexual Health: The Role of Direct and Indirect Communication

**DOI:** 10.3390/ijerph18189760

**Published:** 2021-09-16

**Authors:** Jennifer M. Grossman, Lisette M. DeSouza, Amanda M. Richer, Alicia D. Lynch

**Affiliations:** 1 Wellesley Centers for Women, Wellesley College, Wellesley, MA 02481, USA; ldesouza@wellesley.edu (L.M.D.); aricher@wellesley.edu (A.M.R.); 2 Lynch Research Associates, 41 Oakland Street Ext, Natick, MA 01760, USA; aliciadlynch@gmail.com

**Keywords:** reproductive health, adolescence, family communication, father, sexual behavior, indirect talk

## Abstract

Family talks about sex can protect against teens’ risky sexual behavior, but most research has focused on the role of mothers. The current study included cross-sectional survey data from 728 adolescents in the 11th and 12th grades (M_age_ = 17.00, SD = 0.90) in the United States. Structural equation modeling (SEM) was used to assess associations between teens’ direct and indirect talk, defined as less straightforward ways to communicate one’s sexual values, with fathers about sex, and teens’ sexual behaviors. There were no significant direct associations between father-teen talk about sex and teens’ sexual behavior. However, teen gender moderated associations between indirect father-teen communication and teens’ sexual behavior. The results suggest the need to assess indirect talk about sex in studies of family sexuality communication and to further investigate the role of teens’ identities in determining the influence of father-teen talk about sex on teens’ sexual behavior.

## 1. Introduction

Risky sexual behaviors, such as early sex and lack of protection, leave teens vulnerable to sexually transmitted infections (STIs) and unplanned pregnancy [1]. The World Health Organization reports that there are more than one million new cases of treatable STIs worldwide each day [2]. In the United States, rates of chlamydia, gonorrhea, and syphilis are increasing [3], and one in four sexually active female teens has an STI [4]. Further, only 40% of teen mothers finish high school [5], incurring educational and economic costs for themselves and their offspring [6]. This risk is particularly high for Black and Latino teens, who are twice as likely to become teen parents as White adolescents [7].

Sex education programs can reduce teens’ sexual risk behavior [8,9], but most teens do not have access to school-based comprehensive sex education, with only 13 states mandating that sex education instruction be medically accurate [10]. Parents’ talks with their teens about sex and relationships can help to address this gap in access. These conversations can promote teens’ delay of sex and their use of protection when they do have sex [11,12,13,14].

Sexual socialization theory posits that discussions about sex and relationships provide opportunities for families to share their values and beliefs about sex with their children [15]. Dittus and her colleagues’ conceptual model of mother-teen sexuality communication assumes that not all parent-teen communication about sex is direct [16]. Instead, it includes both direct talks about sex and indirect communication (see definition below). The current study extended Dittus and colleagues’ model to father-teen communication. Research has shown that fathers play an important role in supporting teens’ sexual health, which provides health benefits to teens and young adults over and above support from mothers [17,18]. This suggests that fathers contribute to teens’ sexual health, but also do so in unique ways. However, research has not yet explored how fathers may talk differently than mothers about sex and relationships or how these conversations shape teens’ sexual health.

Findings suggest that mothers are the primary family communicators about sex and relationships [19,20] and most research on parent-teen talks about sex and its effects on teens’ health focuses on mothers [14]. Studies of family talks about sex have often left out fathers altogether or assessed communication with an unspecified “parent” [14,19]. Studies have shown that less than half of fathers talk with their teens about sex [19], but questions remain about how fathers talk with their teens about sexual issues and whether this communication is fully captured in measures of family talk about sex. A growing field of research has begun to explore the roles of fathers in supporting teens’ sexual health. Reviews of research have shown significant protective associations between father-teen talks about sex and teen sexual behavior [18,19]. This research can inform health education programs for teens, which rarely focus on the role of fathers [18]. Expanding research on father-teen talks about sex and teens’ sexual health can help to guide programs that use an understanding of father-teen communication to inform best practices for prevention and intervention.

One question about the role of fathers in their talks with teens about sex and relationships is how they talk with their teens about sex. Talk about sex is typically measured by one or more questions that ask about whether certain topics have been discussed, such as delaying sex, using protection, and sexually transmitted infections [14]. We describe this as “direct” communication about sex. This direct assessment is how most father-teen communication about sex has been measured. This assessment has shown low levels of father-teen talks about sex but has also found that these conversations can be protective against teens’ sexual risk behaviors [18,19].

Indirect talk about sex provides another way to understand and assess how fathers may talk with teens about sex and relationships, which may capture aspects of father-teen communication about sex that are seldom measured. Indirect communication consists of less straightforward ways to communicate one’s sexual values, such as conveying general value systems and social modeling. It includes interactions such as talking with someone else about a sexual issue while a teen is in the room or alluding to sexual behavior without using clear sexual language [21]. This construct has been used to understand communication about sex in Latinx families, given cultural taboos that can inhibit open communication about sex [22,23]. However, most research addressing the cultural reluctance to talk about sexual issues has focused on Latinx mothers [22,24]. For example, a qualitative study of Mexican mothers found that participants often identified shame as a barrier to direct talks with teens about sex [25]. Research is needed to assess indirect talks between fathers and teens about sex, particularly among Latinx families, and whether these talks can be protective for teens’ sexual behavior.

Father-teen communication about sex also needs to address key layers of culture and context. Studies have shown differences in the content, frequency, and effects of parent-teen sexuality communication with sons versus daughters [14,20,26,27]. Findings suggest that parents often struggle to talk with sexual-minority teens about sex [28,29] and results are mixed as to whether the impact of parent-teen talks about sex is the same for sexual minorities compared to heterosexual teens [30,31]. Few studies have assessed the influence of residential versus nonresident fathers on teens’ sexual health [18], although a review confirmed the importance of nonresident fathers in children’s well-being [32].

Research is needed to address gaps in the understanding of father-teen communication about sex, particularly in Latinx families. The assessment of indirect communication provides one avenue that may capture aspects of father-teen communication that are not typically measured in research studies. Associations between indirect talk about sex and teens’ sexual behaviors have rarely been investigated and have not been explored with fathers specifically. Finally, these associations must be understood in relation to key contexts of father-teen communication: namely teens’ gender and fathers’ residential status. This investigation can help to identify whether and in what contexts fathers’ indirect communication with teens may be protective for teens’ sexual health.

This study was unique in its focus on indirect father-teen communication and contextual factors that may shape relationships of father-teen communication about sex and teen sexual behavior. It assessed (1) associations of direct and indirect father-teen talk about sex with teens’ sexual behaviors and (2) whether associations between father-teen talks about sex and teens’ sexual behaviors differ based on teens’ gender and fathers’ residential status. Based on prior research, we hypothesized that direct father-teen talks about sex would be associated with lower levels of sexual activity and sexual risk behavior. We expected stronger associations between father-teen talks about sex and teens’ sexual behavior for male teens and weaker associations for nonresidential fathers. Given the dearth of research on indirect father-teen talk about sex, we did not hypothesize about its associations with teens’ sexual behavior.

## 2. Materials and Methods

### 2.1. Recruitment and Participants

The present research included 11th- and 12th-grade students (M_age_ = 17.00, SD = 0.90) who completed a survey about family talks about sex and relationships. Nine hundred and fifty-two youth completed the survey. The study method and associated protocols were approved by the institution’s Institutional Review Board (19 December 2016). This survey was piloted with high-school students before administration, and had a 6th-grade reading level established by the Flesch-Kincaid Grade Level readability test.

Six urban schools in New England were recruited to participate in this study and offered a USD 500 stipend to support a study liaison and the distribution of information to parents. Four schools opted for a waiver of consent (i.e., passive consent) for teen participation, and two schools selected active parental consent. All materials distributed to parents were translated into commonly spoken home languages in those schools. Further information about study recruitment is available in a prior publication [33].

Before participation, teens were asked to give their assent. Teens completed the survey in classrooms on school computers or tablets provided by the research team. The average time to complete the survey using Qualtrics software was 20–30 min, and students could complete the survey in English or Spanish. Teachers and students were encouraged to maintain privacy during survey completion.

The current study included a subsample of 728 youth (M_age_ = 17.00, SD = 0.90) who reported having a father in their life. The teens self-identified as 53% Latinx, 16% Black, 17% White, 8% Asian, 4% Middle Eastern, and 1% biracial. Self-reported gender was 47% male, 52% female, and 1% neither or unsure of gender. Most students (87%) took the survey in English, while 13% took it in Spanish. Teens reported that 27% of their parents had been teen parents themselves, and 79% of participants reported that one or both of their parents immigrated to the United States. Their mothers’ median education was some training after completion of high school, and their fathers had completed a high school education. See Table 1.

### 2.2. Measures

#### 2.2.1. Direct Communication

Questions about direct communication were only asked if a teen identified that they talked to a father about sex. Direct communication about sex was assessed in three domains. Risks of Sex refers to negative consequences of sex (4 items, α = 0.86), Protection includes how to prevent pregnancy, STIs, and HIV/AIDS (3 items, α = 0.93), and Relational Sex indexes conversations about sex being normal in the context of a trusting relationship (3 items, α = 0.87) [33]. Items were both generated for this study and adapted from measures of parent-teen talks about sex [34,35]. Responses included 1 (“Never”), 2 (“Rarely”), 3 (“Sometimes”), 4 (“Often”), and 5 (“All the time”). A sample item is: “In the past year, how often have your [family member] talked about these topics: the dangers of getting a Sexually Transmitted Disease.” This measure was psychometrically assessed in a prior paper and showed excellent fit statistics (CFI = 0.97, TLI = 0.96, RMSEA = 0.07) [33].

While in past research, direct communication has differentiated into three domains of direct communication about sex when talking with a mother or with an extended family member [33], in the present research, direct communication with fathers about sex represents one global factor (M = 1.80, SD = 1.10, α = 0.96). See Table 2. Psychometric analysis of youth reports of father communication and adolescent sexual behaviors suggested that the three domains of direct communication assessed were best-modeled as one factor. Model fit statistics from a CFA suggest a very good fit for a one-factor model (CFI = 0.97, TLI = 0.95, RMSEA = 0.13).

#### 2.2.2. Indirect Communication

All participants were asked to respond to questions about indirect communication, which refers to less straightforward communication about relationships or sex. This construct was measured using the Indirect Family Sexuality Communication Scale [21], which included three items (M = 1.62, SD =0.80, α = 0.78) on a scale of 1 (“Never”), 2 (“Rarely”), 3 (“Sometimes”), 4 (“Often”), and 5 (“All the time”). A sample item is: “How often does your [family member] do this: make comments to you about the sexual behavior of movie and TV characters.” See Table 2.

#### 2.2.3. Moderating Variables

Two moderating variables were included in the present analyses. Gender was reported as “male,” “female,” “neither male nor female,” or “not sure”, and was coded as female (1) or male (0). No other gender categories had sufficient power to be assessed. To determine fathers’ residential status, teens were asked if they lived with a “mother and father in same place,” “mother and father in different places,” “a mother,” “a father,” “2 mothers in the same place,” “2 mothers in different places,” “2 fathers in the same place,” “2 fathers in different places,” “other relatives, not parents,” or “someone outside of family.” If their response indicated that they lived with a “mother and a father in the same place,” with “a father,” or with “two fathers in the same place,” they were coded as (1); if they did not live with a father, they were coded as (0).

#### 2.2.4. Control Variables

Covariates were included in each model to reduce the possibility of omitted variable bias. Teens’ *race/ethnicity* was reported in racial/ethnic categories (Black, Asian, Latinx, White, Middle Eastern, and Biracial). In this sample, the racial/ethnic groups with sufficient power to be modeled separately were Asian, Black, and Latinx youth, in comparison to White youth. We also accounted for family factors in the covariates, including if the teen communicated about sex with their mother or primary caregiver (1) or did not communicate (0), if either parent was an immigrant to the U.S. (1) or was not an immigrant (0), whether they spoke languages other than English at home (1) or only English (0), and if either parent was a teen parent (1) or was not a teen parent (0). Social desirability was also included as a covariate and was measured with the sum of five items, indicating whether the teen had engaged in a particular behavior (e.g., “I am always polite even to people who are not nice to me;” 1 = True, 0 = False), with higher scores indicating more of an influence of social desirability on youth responses. When not used as a primary moderating variable, gender and father’s residence were additionally included as covariates. We used attraction as a proxy for sexual orientation, which teens reported by indicating if they were attracted to “opposite sex,” “same sex,” “both males and females,” “not sure,” or “neither.” If teens responded with “opposite sex,” they were coded as (1), and if they indicated any other response, they were coded as (0). Given the small number of nonheterosexual teens, this variable was only assessed descriptively.

#### 2.2.5. Outcomes

Participants reported their sexual activity by indicating whether they had ever had oral sex (1) or never had oral sex (0) and if they had ever had vaginal sex (1) or never had vaginal sex (0). If youth had engaged in vaginal sex, they were asked follow-up questions about their recent use of protection and number of partners. Condom use in the last 12 months was measured using a single item on a 5-point scale, 1 = “Never” to 5 = “Always.” Number of vaginal sex partners in the past 12 months was measured continuously (e.g., 3 = 3 partners) and ranged from 1 partner (1) to 6 or more partners (6). Early vaginal sex was determined by the age the participant reported first vaginal sex. If the age of first vaginal sex was before the age of 15, they were coded as (1) early vaginal sex, and sex after age 15 was coded as (0) not early vaginal sex.

### 2.3. Analysis

Structural equation models were used to test associations between direct and indirect communication about sex and youth sexual behavior outcomes (sexual activity, number of partners, early sex, and condom use), controlling for demographic differences. In Step 1 of these analyses, direct effects were assessed between direct and indirect communication and these outcomes. In Step 2, moderated effects were assessed to see if direct and indirect communication interacted with teen gender and father’s residential status. Each SEM included a measurement model and structural model. The measurement model included direct and indirect communication as latent variables (CFI = 0.96, TLI = 0.95, RMSEA = 0.05). The structural model included the predictive relationship between these variables and the sexual behavior outcomes. In the moderation models, observed variables of gender and residential status were also regressed on the youth sexual behavior outcomes. The previously described variables to control for background experiences and demographic differences were included in all models. The models that tested dichotomous outcomes (e.g., early sex) use a weighted least squares estimator, and the models that tested continuous outcomes used full information maximum likelihood (FIML). All analyses were conducted in Mplus Version 7 [36].

## 3. Results

### 3.1. Descriptive Statistics

One third of the teens reported having had vaginal sex (33%) and 37% reported having had oral sex. Of the youth who reported having had vaginal sex, 28% reported having sex before age 15. The average age of sexual debut was 15.35 years (SD = 3.23), the number of partners was 1.76 (SD = 1.37), and condom use was half the time to more than half the time (M = 3.57, SD = 1.55). Forty-five percent of youth in this sample reported having at least some direct communication with their fathers about sex. Fifty-six percent of youth reported indirect communication with their father. Overall, 427 youth (59%) reported some form of communication with fathers about sex. In the total sample, 28% (*n* = 207) reported indirect communication only, 6% (*n* = 43) reported direct communication only, and 24% (*n* = 177) reported both direct and indirect communication. The similar means for direct talk with fathers about sex (M = 1.80, SD = 1.10) and indirect talk with fathers about sex (M = 1.62, SD = 0.80) suggest teens “rarely” communicate with their fathers about sex, on a scale where 1 indicates “never” and 5 indicates “all the time.” The information on percentages and means indicates that close to half of participants reported some direct or indirect talk with fathers about sex, but most participants reported that direct and indirect talks with their fathers were infrequent. Fifty-eight percent (*n* = 126) of male teens and 42% (*n* = 92) of female teens reported direct communication with their fathers, while 61% (*n* = 210) of male teens and 45% (*n* = 169) of female teens reported indirect communication. Forty-seven percent of heterosexual youth (*n* = 199) and 36% (*n* = 20) of nonheterosexual youth reported direct communication with their fathers about sex, while 57% (*n* = 342) of heterosexual youth and 51% (*n* = 40) of nonheterosexual youth reported indirect communication. Finally, 59% of youth with a nonresidential father (*n* = 58) reported direct communication, and 42% of teens reported direct communication with a father (*n* = 162). Teens with a nonresidential father also reported indirect communication (57%, *n* = 77), and 56% (*n* = 307) of teens with a residential father also reported indirect communication. See Table 3.

### 3.2. Structural Equation Models

**Figure 1:**
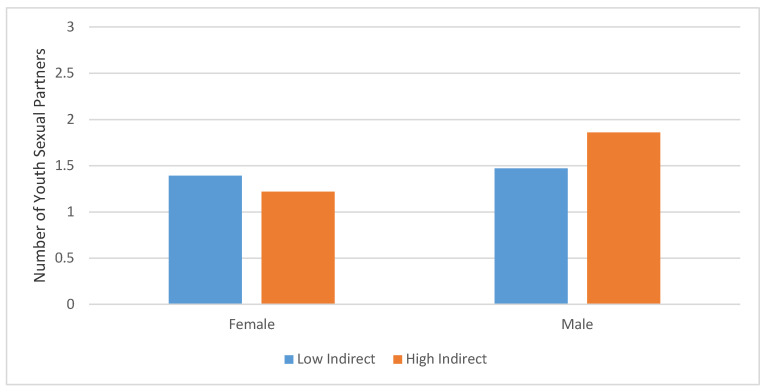
Teen gender as a moderator of the link between father-teen indirect communication and number of sexual partners.

There were no statistically significant direct effects of direct or indirect communication on any of the youth sexual behavior outcomes detected in Step 1 of these analyses. However, direct effects are not necessary to detect moderated effects, so we proceeded with the next phase of the analysis assessing interactions. Gender significantly moderated the link between indirect communication and number of partners (β = −0.23, SE = 0.10, *p* = 0.02), but did not moderate the link between direct communication and number of partners (see Table 4). Adding interaction terms to the model improved the model fit R^2^ = 0.09, *p* = *0*.05. Categorical tests of simple slopes were not significant when fixed at 0 = male (B = 0.28, SE= 0.22, *p* = 0.194) or when fixed at 1 = female (B = −0.32, SE = 0.22, *p* = 0.148). Therefore, while there is a difference in gender, the unique function of communication by gender for each group was not detectable in the present analysis. A graphic representation of the descriptive relationship between high/low indirect communication and gender with number of sexual partners is presented in Figure 1. Descriptively, female teens engaging in lower rates of indirect communication report slightly fewer sexual partners than do female teens who indirectly communicate at higher rates. Conversely, male teens who report more indirect communication with fathers also report more sexual partners than do male teens engaging in lower rates of indirect communication.

Father’s residential status was also tested for direct and moderating effects, but no significant effects were found with direct or indirect communication about sex and sexual outcomes.

## 4. Discussion

This study is one of the first to explore fathers’ indirect communication with teens about sex and the contextual factors that shape its relationship with teens’ sexual behaviors. It is interesting that 56% of youth reported indirect communication with their father, compared to 45% of youth who reported direct communication, despite relatively comparable rates of communication in both domains. This indicates that more fathers may use indirect communication as a strategy to convey expectations and messages about sex to teens, a type of communication that is not typically assessed in sexuality communication research. This finding is consistent with Dittus’ model of mother-teen talks about sex, which includes indirect communication [16], and indicates the need to use expanded indictors to effectively assess father-teen communication about sex. Fathers’ indirect communication may reflect a different style of communication about sex than that typically used by mothers. It may also show fathers’ hesitancy or discomfort with talking with teens about sex, which has been used to explain high rates of indirect parent-teen talks about sex among Latinx families [25,37]. As most programs designed to increase family talks about sex focus on mothers [18], fathers may have fewer tools to talk with their teens about sex and may not view themselves in this role, in part based on the lack of talks with their own fathers about sexual issues [38].

The lack of primary associations of father-teen direct and indirect talk about sex with teens’ sexual behaviors may reflect the low levels of teens’ reported talks with fathers about sex, as well as low levels of teens’ sexual behavior in this sample. The high percentage of Latinx teens in this sample may in part explain low levels of father-teen talks about sex, as talks about sex are often considered taboo in Latinx cultures [23,24]. However, most prior studies of talks with teens about sex in Latinx families were conducted with mothers (e.g., 22,24), so further research on Latinx fathers is needed to address this question. This lack of association may also reflect other influential factors that may enhance the role of communication, as we see when we account for some moderating influences on communication such as gender. However, these findings are inconsistent with prior research that found direct associations between father-teen talks about sex and teens’ sexual behavior [18,19].

The finding that indirect father-teen communication relates to the number of sexual partners fits with prior research that shows that teen gender plays a role in shaping impacts of parents’ talks about sex [14,27]. However, little research has assessed the role of teen gender in fathers’ direct talks with their children. For example, existing studies suggest that fathers may talk more with their sons than daughters about sex, particularly about topics such as condom use [19,39,40], but findings are inconsistent regarding the relationships of this communication with sons’ and daughters’ sexual behaviors [19]. To our knowledge, no studies have assessed how fathers’ indirect talk with teens differs by gender. The positive association between father-teen indirect talk and the number of teens’ sexual partners in the current study may reflect increased father engagement in talks with their teen children about sex once they are perceived to become sexually active. A limitation of the present analysis is that the specific patterns of communication by gender in relation to number of partners was not detected. Descriptive findings suggest that the pattern for male teens is that higher indirect communication may relate to a higher number of sexual partners, and for female teens, higher indirect communication may relate to fewer sexual partners. Future research might offer insight into how conversations differ by gender. In particular, qualitative research that includes fathers, mothers, and teens is needed to explore in-depth family processes and practices regarding talks with teens about sex and the role of parent and teen gender in family talks about sex.

The lack of a moderating role for residential versus nonresidential fathers is surprising, as one would expect that father-teen talks with fathers who live with their teens about sex may have a greater impact on teens’ behaviors than talks with nonresidential fathers given more opportunities to talk with teens about sex and relationships. It may be that fathers who do not live with their teens but still talk with them about sex are those with high engagement and closeness with their teens, which could explain the lack of moderation. In one of the only studies of sexual socialization of teens among residential and nonresidential fathers, findings showed similar reported topics discussed by both groups [40].

Descriptively, nonheterosexual youth reported more indirect than direct communication with fathers. Given that parents often feel uncomfortable talking with sexual-minority teens about sex and relationships [28,29], indirect talk may provide an additional way for fathers and teens to communicate about sexual issues that may be more accessible for some families. Indirect talk about sex, such as tals about teens’ friends or other family members, may avoid potentially awkward or conflictual conversations related to a teen’s potential sexual orientation, but still provide ways for parents and teens to convey information and values about sex and relationships. Given previous findings for lower levels of parent-teen talks about sexual issues with sexual-minority teens compared to heterosexual teens [41] and less talks with fathers about the risks of sex for sexual-minority teens than heterosexual teens [42], further investigation of direct and indirect talk may help to clarify and expand the picture of family communication about sex for this group.

Key limitations of this study include its cross-sectional sample, which does not allow for causal inferences, the low level of sexual activity for participating teens compared to national norms [43], the small number of teens who identified as nonheterosexual, and a lack of qualitative data to give context to the findings. Over half of this study’s sample self-identified as Latinx, which provides valuable data on Latinx fathers. However, more research is needed to disentangle how components of culture and gender interact to shape father-teen talks about sex in Latinx families. The investigation of father-teen direct and indirect talk about sex is also needed among families from other racial/ethnic groups, such as Asian American families, whose communication with teens about sex may follow different patterns than other groups [44,45]. Further, constructs of direct and indirect talk about sex are complex and multi-faceted and the included measures may not capture all aspects of these constructs. In addition, anal sex was not assessed in this study, which limits this study’s findings to vaginal and oral sex.

## 5. Conclusions

This study highlights the role of indirect communication in father-teen talks and raises new questions about the influence of family and teen contexts in shaping its impact. It highlights the need to measure multiple modes of talk about sex in more expanded ways that may better reflect how fathers talk with teens about sexual issues. Qualitative research that explores how teens understand and experience indirect talk in their families about sex may help to clarify these quantitative results. Given the identified role of indirect mother-teen talk about sex in Latinx families [21,22] and the finding that over half of the current majority-Latinx sample reported indirect communication with their fathers about sex, future research would benefit from the exploration of father-teen indirect talk about sex, specifically within Latinx samples. Findings comparing mothers’ and fathers’ talks with teens about sex would provide a more complete picture if they included indirect, as well as direct, communication. Longitudinal studies are also needed to understand the directionality of associations between father-teen direct and indirect communication and teens’ sexual behavior, such as whether parent-teen talks about sex come before or after teens become sexually active. Qualitative studies that have explored interactions between mothers’ and fathers’ communication with teens about sex and how parents communicate about these roles could also help to illuminate these relationships. As a growing body of research has shown fathers’ impacts on their teens’ health, there is a need to focus more research on father-teen talks about sex, and to address the lack of health education programs that specially engage fathers. Research is needed to understand how fathers talk with teens about sex, whether and how this talk differs from mothers’ talks with teens in content and process, and what resources fathers want and need to support their communication with teens about sex and relationships. This research, in turn, can inform programs that are designed to support and engage fathers in health-promoting sexuality communication with their teens.

## Figures and Tables

**Table 1:** Sample descriptive statistics.

	Analysis Sample (*n* = 728)		Male (*n* = 341)		Female (*n* = 378)	
	M(SD) or %	*n*	M(SD) or %	*n*	M(SD) or %	*n*
Female	52%	728	--	--	--	--
Male	47%	728	--	--	--	--
Neither male nor female/Not sure	1%	728	--	--	--	--
Black	16%	728	18%	339	14%	376
Latinx	53%	728	55%	339	53%	376
Asian	8%	728	7%	339	9%	376
White	17%	728	16%	339	17%	376
Middle Eastern	4%	728	3%	339	6%	376
Biracial	1%	728	2%	339	1%	376
Child of Teen Parent	27%	557	24%	239	30%	314
Parent Immigration	79%	684	77%	316	82%	361
Language Spoken (English)	87%	643	87%	291	87%	345
Social Desirability	2.71(1.04)	639	2.70(1.08)	289	2.74(1.02)	343

Note: Not all students completed each question, resulting in missing data on some variables. Sub-group n’s may not add up to total n’s, due to data for youth who did not identify in Male or Female categories.

**Table 2:** Direct and indirect communication items and standardized factor loadings.

Direct Communication	Loading
Protecting yourself from STDs	0.88
Protecting yourself from the HIV/AIDS virus	0.89
Protecting yourself from becoming pregnant or getting someone pregnant	0.88
Symptoms of sexually transmitted diseases	0.79
The dangers of getting a sexually transmitted disease	0.89
The negative things that would happen if you got pregnant or if you got someone pregnant	0.81
Sex is ok if both people agree to it	0.78
It’s ok to have sex if it’s with someone special to you	0.76
Being sexual is a natural part of being human	0.75
**Indirect Communication**	
Make comments to you about other people’s sexual behavior	0.86
Talk to other people about sexual issues when you are in the room	0.72
Make comments to you about the sexual behavior of movie and TV characters	0.72

Note: All factor loadings significant at *p* < 0.001. CFI = 0.96, TLI = 0.95, RMSEA = 0.05.

**Table 3:** Descriptive statistics for study measures.

	Analysis Sample (*n* = 728)		Male (*n* = 341)		Female (*n* = 378)	
	M(SD) or %	*n*	M(SD) or %	*n*	M(SD) or %	*n*
Heterosexual Attraction	89%	700	93%	324	86%	368
Talk with a Mother	60%	706	52%	325	67%	373
Direct Comm	1.81(1.10)	486	1.90 (1.11)	258	1.71(1.09)	222
Indirect Comm	1.62(.80)	683	1.78(.82)	314	1.48(.76)	363
Had Oral Sex	37%	693	42%	324	32%	631
Had Vaginal Sex	33%	693	38%	324	29%	362
Early Sex (before 15)	28%	224	33%	120	19%	99
Number of Sexual Partners (past 12 months)	1.76(1.37)	200	1.93(1.46)	99	1.46(1.02)	96
Condom Use (past 12 months)	3.57(1.55)	211	3.75(1.47)	108	3.42(1.58)	98

Note: Not all students completed each question, resulting in missing data on some variables. Sub-group n’s may not add up to total n’s, due to data for youth who did not identify in Male or Female categories.

**Table 4:** Moderating effects on communication about sex and number of partners.

	Number of Sex Partners (Direct Effects) R^2^ = 0.05, *p* = 0.115 CFI = 0.88, TLI = 0.85, RMSEA = 0.06						Number of Sex Partners (Gender Moderator) R^2^ = 0.09, *p* = 0.055					
	B	SE	*p*	β	SE	*p*	B	SE	*p*	β	SE	*p*
Indirect Comm	0.05	0.16	0.739	0.04	0.12	0.738	0.28	0.22	0.194	0.22	0.17	0.188
Direct Comm	0.07	0.18	0.702	0.05	0.14	0.701	−0.01	0.19	0.956	−0.01	0.15	0.956
Indirect Comm X Moderator	--	--	--	--	--	--	−0.60	0.28	0.033	−0.23	0.10	0.024
Direct Comm X Moderator	--	--	--	--	--	--	0.13	0.27	0.623	0.05	0.11	0.625
Female	0.28	0.20	0.159	0.11	0.08	0.142	0.28	0.19	0.135	0.11	0.07	0.119
Heterosexual Attraction	0.10	0.27	0.715	0.03	0.07	0.712	0.05	0.25	0.849	0.01	0.07	0.848
Black	−0.09	0.40	0.820	−0.03	0.12	0.820	0.02	0.40	0.967	0.01	0.12	0.967
Latinx	−0.20	0.34	0.558	−0.08	0.13	0.558	−0.09	0.34	0.785	−0.04	0.13	0.785
Asian	−0.59	0.35	0.090	−0.13	0.08	0.080	−0.45	0.33	0.168	−0.10	0.07	0.159
Child of Teen Parent	−0.23	0.20	0.244	−0.08	0.07	0.237	−0.22	0.20	0.272	−0.08	0.07	0.267
Talk with Mother	−0.01	0.25	0.969	0.00	0.10	0.969	−0.01	0.24	0.964	0.00	0.09	0.964
Language Spoken	0.28	0.20	0.148	0.07	0.05	0.135	0.32	0.20	0.113	0.08	0.05	0.095
Parent Immigration	−0.07	0.30	0.806	−0.02	0.10	0.805	−0.10	0.29	0.722	−0.03	0.09	0.721
Social Desirability	−0.01	0.10	0.879	−0.01	0.08	0.878	−0.01	0.09	0.905	−0.01	0.08	0.905
Residential Father	0.04	0.19	0.826	0.01	0.06	0.826	0.01	0.18	0.938	0.01	0.06	0.938

## Data Availability

Data for this project are archived at the InterUniversity Consortium for Political and Social Science Research (ICPSR).

## References

[1] Kann L., McManus T., Harris W.A., Shanklin S.L., Flint K.H., Queen B., Lowry R., Chyen D., Whittle L., Thornton J. (2018). Youth Risk Behavior Surveillance—United States, 2017. MMWR. Surveill. Summ..

[2] World Health Organization More than 1 Million New Curable Sexually Transmitted Infections Every Day 2019. https://www.who.int/news/item/06-06-2019-more-than-1-million-new-curable-sexually-transmitted-infections-every-day.

[3] Centers for Disease Control and Prevention. Division of STD Prevention, National Center for HIV/AIDS, Viral Hepatitis, STD, and TB Prevention, Centers for Disease Control and Prevention National Overview of STDs 2019. https://www.cdc.gov/std/statistics/2019/overview.htm#Chlamydia.

[4] Forhan S.E., Gottlieb S.L., Sternberg M.R., Xu F., Datta S.D., McQuillan G.M., Berman S.M., Markowitz L.E. (2009). Prevalence of Sexually Transmitted Infections Among Female Adolescents Aged 14 to 19 in the United States. Pediatrics.

[5] Shuger L. (2012). Teen Pregnancy and High School Dropout: What Communities are Doing to Address These Issues.

[6] Westman J. (2009). Breaking the Adolescent Parent Cycle: Valuing Fatherhood and Motherhood.

[7] Centers for Disease Control and Prevention Natality Public-Use Data 2007–2017. http://wonder.cdc.gov/natality-current.html.

[8] Denford S., Abraham C., Campbell R.M., Busse H. (2016). A comprehensive review of reviews of school-based interventions to improve sexual-health. Health Psychol. Rev..

[9] Lameiras-Fernández M., Martínez-Román R., Carrera-Fernández M.V., Rodríguez-Castro Y. (2021). Sex Ed-ucation in the Spotlight: What Is Working? Systematic Review. Int. J. Environ. Res. Public Health.

[10] Guttmacher Institute (2016). Sex and HIV Education. State Policies in Brief. https://www.guttmacher.org/statecenter/spibs/spib_SE.pdf.

[11] Akers A.Y., Holland C.L., Bost J. (2011). Interventions to Improve Parental Communication About Sex: A Systematic Review. Pediatrics.

[12] Bastien S., Kajula L.J., Muhwezi W.W. (2011). A review of studies of parent-child communication about sexuality and HIV/AIDS in sub-Saharan Africa. Reprod. Health.

[13] Sutton M.Y., Lasswell S.M., Lanier Y., Miller K.S. (2014). Impact of parent-child communication interven-tions on sex behaviors and cognitive outcomes for Black/African-American and Hispanic/Latino Youth: A systematic review, 1988–2012. J. Adolesc. Health.

[14] Widman L., Choukas-Bradley S., Noar S.M., Nesi J., Garrett K. (2016). Parent-Adolescent Sexual Com-munication and Adolescent Safer Sex Behavior: A Meta-Analysis. JAMA Pediatr..

[15] Shtarkshall R.A., Santelli J.S., Hirsch J.S. (2007). Sex Education and Sexual Socialization: Roles for Educators and Parents. Perspect. Sex. Reprod. Health.

[16] Dittus P.J., Jaccard J., Gordon V.V. (1999). Direct and nondirect communication of maternal beliefs to ado-lescents: Adolescent motivations for premarital sexual activity. J. Appl. Soc. Psychol..

[17] Grossman J.M., Black A.C., Richer A.M. (2020). Combination of Parent-Child Closeness and Parent Disapproval of Teen Sex Predicts Lower Rates of Sexual Risk for Offspring. J. Fam. Issues.

[18] Guilamo-Ramos V., Bouris A., Lee J., McCarthy K., Michael S.L., Pitt-Barnes S., Dittus P. (2012). Pa-ternal influences on adolescent sexual risk behaviors: A structured literature review. Pediatrics.

[19] Wright P.J. (2009). Father-Child Sexual Communication in the United States: A Review and Synthesis. J. Fam. Commun..

[20] Scull T.M., Carl A.E., Keefe E.M., Malik C.V. (2021). Exploring Parent-gender Differences in Parent and Adolescent Reports of the Frequency, Quality, and Content of Their Sexual Health Communication. J. Sex Res..

[21] Raffaelli M., Green S. (2003). Parent-Adolescent Communication About Sex: Retrospective Reports by Latino College Students. J. Marriage Fam..

[22] Guilamo-Ramos V., Dittus P., Jaccard J., Goldberg V., Casillas E., Bouris A. (2006). The Content and Process of Mother--Adolescent Communication about Sex in Latino Families. Soc. Work. Res..

[23] Murphy-Erby Y., Stauss K., Boyas J., Bivens V. (2011). Voices of Latino parents and teens: Tailored strate-gies for parent-child communication related to sex. J. Child. Poverty.

[24] Alcalde M.C., Quelopana A.M. (2013). Latin American immigrant women and intergenerational sex education. Sex Educ..

[25] Moncloa F., Wilkinson-Lee A.M., Russell S.T. (2010). Cuídate Sin Pena:Mexican Mother-Adolescent Sexuality Communication. J. Ethn. Cult. Divers. Soc. Work..

[26] Kapungu C.T., Baptiste D., Holmbeck G., McBride C., Robinson-Brown M., Sturdivant A., Crown L., Paikoff R. (2010). Beyond the “Birds and the Bees”: Gender Differences in Sex-Related Communication Among Urban African-American Adolescents. Fam. Process..

[27] Zimmer-Gembeck M.J., Helfand M. (2008). Ten years of longitudinal research on U.S. adolescent sexual be-havior: Developmental correlates of sexual intercourse, and the importance of age, gender and ethnic back-ground. Dev. Rev..

[28] Grafsky E.L., Hickey K., Nguyen H.N., Wall J.D. (2018). Youth Disclosure of Sexual Orientation to Siblings and Extended Family. Fam. Relations.

[29] LaSala M.C. (2014). Condoms and Connection: Parents, Gay and Bisexual Youth, and HIV Risk. J. Marital. Fam. Ther..

[30] Bouris A., Hill B.J., Fisher K., Erickson G., Schneider J.A. (2015). Mother-Son Communication About Sex and Routine Human Immunodeficiency Virus Testing Among Younger Men of Color Who Have Sex with Men. J. Adolesc. Health.

[31] Thoma B.C., Huebner D.M. (2014). Parental Monitoring, Parent-Adolescent Communication About Sex, and Sexual Risk Among Young Men Who Have Sex with Men. AIDS Behav..

[32] Adamsons K., Johnson S.K. (2013). An updated and expanded meta-analysis of nonresident fathering and child well-being. J. Fam. Psychol..

[33] Grossman J.M., Lynch A.D., Richer A.M., DeSouza L.M., Ceder I. (2019). Extended-Family Talk about Sex and Teen Sexual Behavior. Int. J. Environ. Res. Public Health.

[34] Sales J.M., Milhausen R.R., Wingood G.M., DiClemente R.J., Salazar L.F., Crosby R.A. (2008). Valida-tion of a parent-adolescent communication scale for use in STD/HIV prevention interventions. Health Educ. Behav..

[35] Eisenberg M.E., Sieving R.E., Bearinger L.H., Swain C., Resnick M.D. (2006). Parents’ Communication with Adolescents About Sexual Behavior: A Missed Opportunity for Prevention?. J. Youth Adolesc..

[36] Muthén L.K., Muthén B.O. (2012). Mplus User’s Guide.

[37] Raffaelli M., Ontai L.L. (2001). ’She’s 16 years old and there’s boys calling over to the house’: An exploratory study of sexual socialization in Latino families. Cult. Health Sex..

[38] Randolph S.D., Coakley T., Shears J., Thorpe R.J. (2017). African-American fathers’ perspectives on fa-cilitators and barriers to father-son sexual health communication. Res. Nurs. Health.

[39] Sneed C.D., Somoza C.G., Jones T., Alfaro S. (2013). Topics discussed with mothers and fathers for parent-child sex communication among African-American adolescents. Sex. Educ..

[40] Sneed C.D., Willis L.A. (2016). Differences between residential and non-residential fathers on sexual socialisa-tion of African American youth. Sex. Educ..

[41] Friedman C.K., Morgan E.M. (2008). Comparing Sexual-Minority and Heterosexual Young Women’s Friends and Parents as Sources of Support for Sexual Issues. J. Youth Adolesc..

[42] Charmaraman L., Grossman J.M., Richer A.M. (2021). Same-Sex Attraction Disclosure and Sexual Commu-nication Topics within Families. J. GLBT Fam. Stud..

[43] Centers for Disease Control and Prevention (2020). Youth Risk Behavior Surveillance—United States, 2019. MMWR Suppl..

[44] Kim J.L., Ward L.M. (2007). Silence Speaks Volumes: Parental Sexual Communication Among Asian Ameri-can Emerging Adults. J. Adolesc. Res..

[45] Trinh S.L., Ward L.M., Day K., Thomas K., Levin D. (2013). Contributions of Divergent Peer and Parent Sexual Messages to Asian American College Students’ Sexual Behaviors. J. Sex Res..

